# Construction of Co-Modified MXene/PES Catalytic Membrane for Effective Separation and Degradation of Tetracycline Antibiotics in Aqueous Solutions

**DOI:** 10.3390/molecules29214995

**Published:** 2024-10-22

**Authors:** Xiaojie Cheng, Xiaojun Qin, Runxue Zhao, Jiamin Chen, Xia Zheng, Ke Liu, Meixuan Xin

**Affiliations:** 1 College of Life Science, Sichuan Normal University, Chengdu 610101, China; f872864996@163.com (R.Z.); eidolonamaz@outlook.com (J.C.); 2 College of Materials and Chemistry & Chemical Engineering, Chengdu University of Technology, Chengdu 610059, China; xjqin913@163.com (X.Q.); zhengxia@stu.cdut.edu.cn (X.Z.); 2023020668@stu.cdut.edu.cn (K.L.); mango_xinmeixuan@163.com (M.X.)

**Keywords:** MXene nanosheets, Co atom catalysts, PMS activation, antibiotic degradation, water treatment

## Abstract

The application of antibiotics has advanced modern medicine significantly. However, the abuse and discharge of antibiotics have led to substantial antibiotic residues in water, posing great harm to natural organisms and humans. To address the problem of antibiotic degradation, this study developed a novel catalytic membrane by depositing Co catalysts onto MXene nanosheets and fabricating the polyethersulfone composite (Co@MXene/PES) using vacuum-assisted self-assembly. The dual role of MXene as both a carrier for Co atoms and an enhancer of interlayer spacing led to improved flux and catalytic degradation capabilities of the membrane. Experimental results confirmed that the Co@MXene/PES membrane effectively degraded antibiotics through peroxymonosulfate activation, achieving up to 95.51% degradation at a cobalt concentration of 0.01 mg/mL. The membrane demonstrated excellent antibacterial properties, minimal flux loss after repeated use, and robust anti-fouling performance, making it a promising solution for efficient antibiotic removal and stable water treatment.

## 1. Introduction

Antibiotics, recognized as emerging pollutants, pose substantial threats to the environment and living organisms due to their persistence, toxicity, and potential for bioaccumulation [1]. These substances and their metabolites not only inhibit the growth of aquatic organisms, but also cause mortality [2]. Furthermore, the emergence of antibiotic-resistant pathogens and the accumulation of residual antibiotics in drinking water present an increasingly severe risk to human health [3,4,5]. Common wastewater treatment methods currently include coagulation [6], adsorption [7], ion exchange [8], activated sludge processes [9], and biological contact oxidation [10]. However, these methods are limited by factors including treatment efficiency, cost, potential secondary pollution during processing, and the inability to fully degrade antibiotics. Membrane separation technologies offer a promising alternative for treating antibiotic-contaminated wastewater due to their mild operating conditions, without the involvement of chemical reagents and high environmental efficiency [11,12]. However, traditional membrane materials are limited by the trade-off effect between selectivity and permeability [13,14]. Additionally, microfiltration and ultrafiltration membranes have pore sizes that are considerably larger than antibiotic molecules, making them ineffective for removing these small molecular pollutants. Although nanofiltration and reverse osmosis membranes demonstrate good separation performance, they suffer from poor permeability, high operational pressures, increased energy consumption, and challenging issues related to membrane fouling.

Since the seminal report by Andre Geim and Konstantin Novoselov on two-dimensional graphene oxide membranes, published in Nature [15], two-dimensional membrane materials have emerged as a prominent area of research [16]. The stacking of two-dimensional nanosheets enables the construction of confined mass transfer channels, facilitating the separation of molecules and ions at the nanoscale. MXenes, an emerging category of two-dimensional materials, possess advantageous properties for membrane fabrication, such as adjustable interlayer spacing and favorable hydrophilicity. Due to the abundant surface functional groups, large specific surface area and excellent conductivity, MXene has been applied in the catalytic fields such as photocatalysis and electrocatalysis, which can be used as both catalyst and carrier for pollutants removal. Wang’s team [17] utilized an ion intercalation strategy to fabricate Al^3+^ intercalated MXene membranes with high antibiotic rejection via vacuum-assisted self-assembly. Additionally, Li et al. [18] demonstrated that pure MXene membranes can achieve a tetracycline removal rate of up to 91.5%. However, small molecular pollutants, including antibiotics, tend to accumulate on the membrane surface and within the interlayer channels of MXene membranes, resulting in membrane fouling [19,20]. Additionally, the narrow interlayer spacing limits the membrane flux.

Advanced oxidation processes degrade organic pollutants into non-toxic small molecules through the generation of highly oxidative free radical [21,22]. Peroxymonosulfate (PMS) can decompose to produce strong oxidizing sulfate radicals (SO_4_^•−^) and hydroxyl radicals (^•^OH) [23]. In addition, free diffused ^•^OH, the surface-bound ^•^OH, SO_4_^•−^ and O_2_^•−^ will also participate in the reaction, which are advantageous for the long-term catalytic degradation of antibiotics [24,25]. Common heterogeneous catalysts include iron-based [26,27], cobalt-based [28], and manganese-based catalysts [29]. For example, cobalt ferrite (CoFe_2_O_4_) has been shown to effectively catalyze PMS activation for antibiotic removal [30]. However, traditional catalysts suffer from low efficiency in utilizing active sites, limiting their effectiveness in antibiotic removal. The use of single-atom catalysts can maximize the utilization of active sites [31], thereby achieving high catalytic efficiency. Nonetheless, catalysts for PMS activation lack support materials, making recovery of the catalysts after pollutant degradation difficult, which hinders their further application in antibiotic treatment.

Based on this, the study deposits Co catalysts onto MXene to create a composite material, which is then utilized to fabricate a polyethersulfone catalytic membrane (Co@MXene/PES) through vacuum-assisted self-assembly. MXene acts as a carrier for Co atoms, which not only expands the interlayer spacing of MXene, enhancing flux, but also imparts catalytic degradation capabilities to the membrane. This dual functionality improves both pollutant removal efficiency and the membrane’s resistance to fouling. The novel Co@MXene/PES catalytic membrane represents a promising strategy for the efficient removal of antibiotic contaminants from aqueous solutions.

## 2. Results and Discussion

### 2.1. Material Characterization

**Figure 1:**
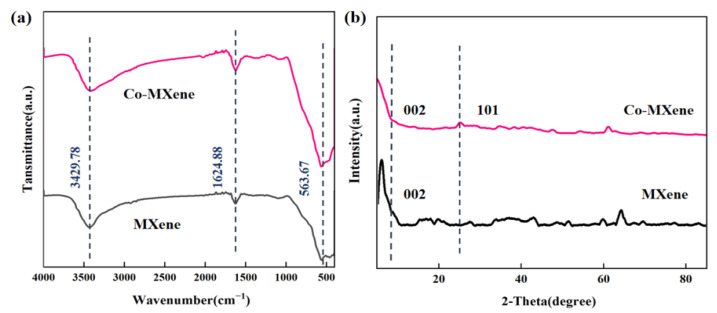
(**a**) FTIR spectra of MXene and Co-MXene; (**b**) XRD patterns of MXene and Co-MXene.

Fourier-transform infrared spectroscopy (FTIR) was employed to identify the types of functional groups within MXene-based materials, which is shown in Figure 1a. Both materials display an -OH stretching vibration peak at 3429.78 cm^−1^ [32]. Additionally, a vibration peak corresponding to C=O or C=C is observed at 1624.88 cm^−1^ [33]. The presence of several low-intensity peaks in the 400–800 cm^−1^ range indicate that the NaOH treatment has increased the oxygen-containing groups on the MXene surface (Figure S1, Supplementary Materials). A prominent absorption peak at 563.67 cm^−1^ in Co-MXene is attributable to the formation of Co-O bonds between the oxygen atoms from NaOH-treated MXene and cobalt [34]. X-ray diffraction (XRD) was used to analyze the crystal structure of MXene and Co-MXene materials. As shown in Figure 1b, the MXene spectrum exhibits a characteristic peak at 2θ = 5.7°, corresponding to the (002) crystal plane [35]. In Co-MXene, this (002) peak shifts to a lower angle (2θ = 5°), indicating an increase in the interlayer spacing of MXene. Additionally, peaks at 2θ = 25.12° and 2θ = 25.45° correspond to the (101) plane in TiO_2_ structure, which come from the oxidation of MXene nanosheets, confirming the successful introduction of oxygen-containing groups. However, no distinct peaks for Co or CoO are observed in the 2θ = 30–45° range, likely due to the low doping level of Co. This suggests that further characterization is required to confirm the successful doping of Co.

**Figure 2:**
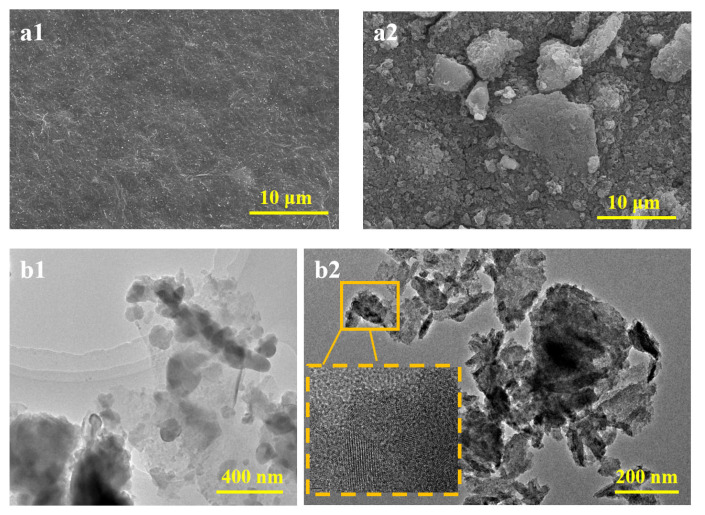
SEM images of pure MXene (**a1**) and the optimal ratio of Co-MXene (**a2**); TEM images of pure MXene (**b1**) and the optimal ratio of Co-MXene (**b2**).

Scanning electron microscopy (SEM) and transmission electron microscopy (TEM) were employed to observe the morphology and structure of the nanomaterials and composite membranes. Figure 2a1,a2 display the SEM images of pure MXene and Co-MXene. MXene shows irregular particle shapes with a dense stacked structure and loose interlayer stacking [16]. In contrast, Co-MXene exhibits a layered structure with significantly increased surface roughness. In addition, Table S1 (Supplementary Materials) shows the elemental composition of Co-MXene nanocomposite, confirming the successful doping of Co atoms on MXene nanosheets. Figure 2b1,b2 show TEM images of pure MXene and Co-MXene materials. It is evident that the surface of MXene is relatively smooth, with the presence of single or multiple layers. In contrast, Co-MXene exhibits significant stacking, and the incorporation of cobalt results in more pronounced lattice fringes and a more complex crystal structure.

### 2.2. Membrane Characterization

**Figure 3:**
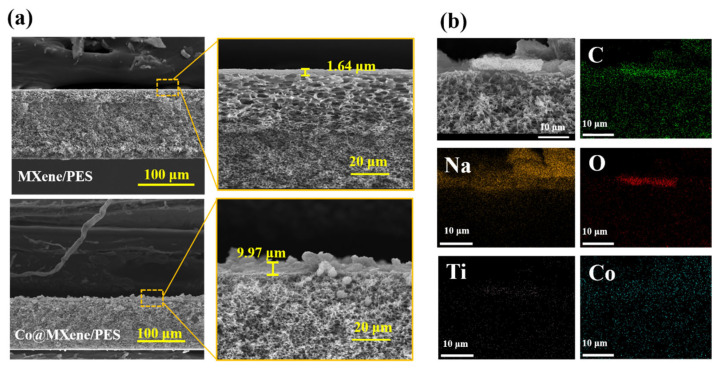
(**a**) Cross-sectional SEM images of pure MXene membrane and the optimal ratio Co@MXene/PES membrane; (**b**) EDS mapping of the optimal ratio Co@MXene/PES membrane.

As shown in Figure 3a, the SEM images of the pure MXene membrane reveal a typical smooth surface with a uniform membrane structure [36]. In contrast, the SEM images of the Co@MXene/PES membrane exhibit significantly increased roughness. At the same mass (12 mg), the Co@MXene/PES membrane has a thickness of 9.97 μm, which is thicker than the pure MXene membrane (1.64 μm). This increased thickness is ascribed to the doping of cobalt, which expands the interlayer spacing of the two-dimensional membrane. Figure 3b illustrates the elemental composition of the Co@MXene/PES membrane. It is evident that, besides the elements C, O, and Ti inherent to MXene, both Na (which is minimally incorporated during the alkali treatment) and Co elements are uniformly distributed throughout the membrane. This distribution confirms that cobalt has been successfully doped into the MXene structure.

**Figure 4:**
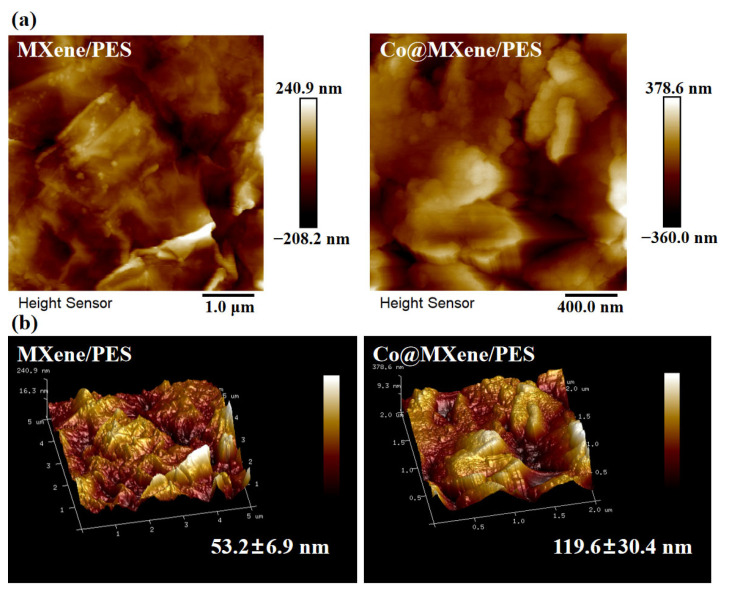
(**a**) 2D AFM diagram of pure MXene membrane and the optimal ratio Co@MXene/PES membrane; (**b**) 3D AFM plot of pure MXene membrane and the optimal ratio Co@MXene/PES membrane.

Atomic force microscopy (AFM) was used to characterize the surface roughness of the composite membranes. As shown in Figure 4, the images indicate that the Co@MXene/PES membrane has an average surface roughness of 119.6 nm, making its surface rougher compared to the pure MXene membrane. This finding is consistent with the SEM observations and indicates that the addition of cobalt increases the membrane’s filtration surface area and hydrophilicity. Additionally, the increased surface roughness exposes more active sites, which can more effectively capture, retain, and interact with contaminants.

The chemical composition and electronic structure of the composite membrane were analyzed using X-ray photoelectron spectroscopy (XPS), which is shown in Figure S2 (Supplementary Materials). The appearance of the characteristic peaks of element Ti indicated the successful construction of the MXene membrane, and element Co exhibited weaker characteristic peak due to its low content. The deconvolution N1s spectra revealed the presence of pyridinic N (400.09 eV) in M3, which was believed to lower the adsorption barrier of PMS on the carbon substrate, thus facilitating the electron transfer and improving the catalytic performance [37]. The Ti2p spectra was fitted as Ti-O 2p_1/2_ and Ti-O 2p_3/2_, corresponding to bond energies of 464.16 eV and 458.50 eV. The Co2p spectrum exhibited characteristic peaks of 796.88 eV and 781.68 eV corresponding to 2p_3/2_ and 2p_1/2_ of Co, respectively [38].

### 2.3. Membrane Permeability and Separation

**Figure 5:**
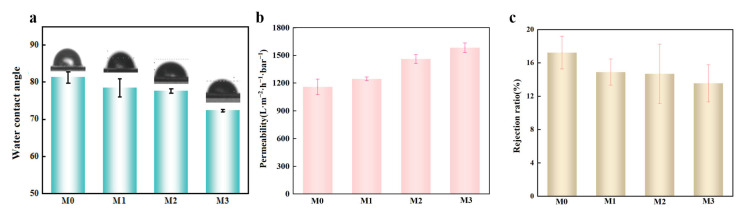
(**a**) The water contact angle of M0–M3; (**b**) Permeability of M0–M3; (**c**) M0–M3 pure retention for TC.

The water contact angle of the membrane reflects its hydrophilicity. As shown in Figure 5a, the contact angle decreases from M0 to M3, indicating that the pure membrane has lower hydrophilicity, which improves gradually with increasing Co content. Figure 5b demonstrates that the membrane permeability increases from 1159.62 L·m^−2^·h^−1^·bar^−1^ (M0) to 1584.60 L·m^−2^·h^−1^·bar^−1^ (M3) as the Co content rises. This increase is attributed to the enhanced surface roughness resulting from Co doping, which improves both hydrophilicity and permeability. Figure 5c shows the rejection rates of the membranes, which decrease from 17.23% (M0) to 13.55% (M3). This reduction is attributed to Co causing partial clogging of the membrane pores, which slightly reduces its separation performance, though the impact is minimal.

### 2.4. Catalytic Membrane Coupled with PMS for TC Removal

#### 2.4.1. Removal Efficiency of Different Membranes

**Figure 6:**
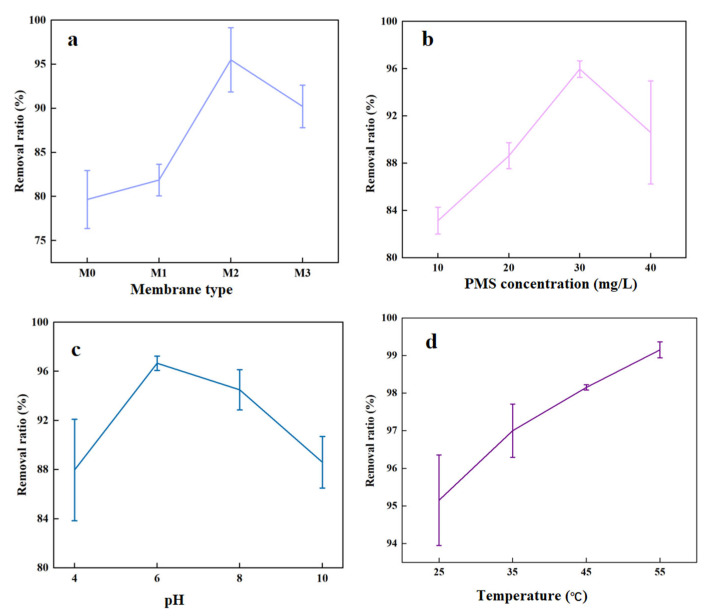
(**a**) Removal rate of TC by different membranes; (**b**) Effect of PMS concentration on TC removal rate (pH = 6, T = 55 °C); (**c**) Effect of pH value on TC removal rate (PMS concentration = 30 mg, T = 55 °C); (**d**) Effect of temperature on TC removal rate (PMS concentration = 30 mg, pH = 6).

To investigate the TC removal efficiency with catalytic membranes coupled with PMS, Figure 6a shows that the pure MXene membrane achieves a removal rate of 79.67%. This rate increases with cobalt content due to the catalytic effect of Co in PMS reduction reactions, which generates free radicals that effectively degrade pollutants. The removal efficiency reaches a peak of 95.51% at a Co content of 0.01 mg/mL but decreases subsequently as Co atoms obstruct membrane pores. Therefore, the membrane with Co content of 0.01 mg/mL (M2) exhibits the highest removal efficiency.

#### 2.4.2. Effect of PMS Concentration on TC Removal Efficiency

Figure 6b shows the effect of PMS concentration on TC removal using the optimal membrane. With other conditions held constant, the TC removal rate increases with PMS concentration up to 30 mg, achieving a peak removal rate of 95.97%. However, further increases in PMS concentration led to a decline in removal efficiency. This is because at lower PMS concentrations, the generated free radicals effectively oxidize TC. At higher PMS concentrations, excess free radicals participate in quenching reactions, reducing their number and significantly impeding the oxidation process [39].

#### 2.4.3. Effect of pH on TC Removal Efficiency

Under optimal conditions for the membrane and PMS, Figure 6c shows the effect of different pH levels on TC removal. The highest TC removal rate of 96.67% is observed at pH = 6. This is attributed to acidic conditions enhancing the activation of PMS to generate SO_4_^•−^, which can effectively degrade TC. In alkaline conditions, PMS activation predominantly produces ·OH or other intermediates, which are less efficient at oxidizing TC compared to SO_4_^•−^. However, very low pH can also limit PMS decomposition rates, reduce radical stability, and induce other side reactions, thereby decreasing the efficiency of oxidation [40].

#### 2.4.4. Effect of Temperature on TC Removal Efficiency

Figure 6d shows that TC removal efficiency increases with temperature, reaching 99.15% at 55 °C under optimal conditions. This improvement is attributed to higher temperatures accelerating the activation of PMS, which increases the generation rate of SO_4_^•−^. Additionally, increased temperatures enhance the interactions between the membrane’s active sites and the contaminants, collectively leading to more efficient TC removal.

### 2.5. Antibacterial Properties of the Catalytic Membrane

**Figure 7:**
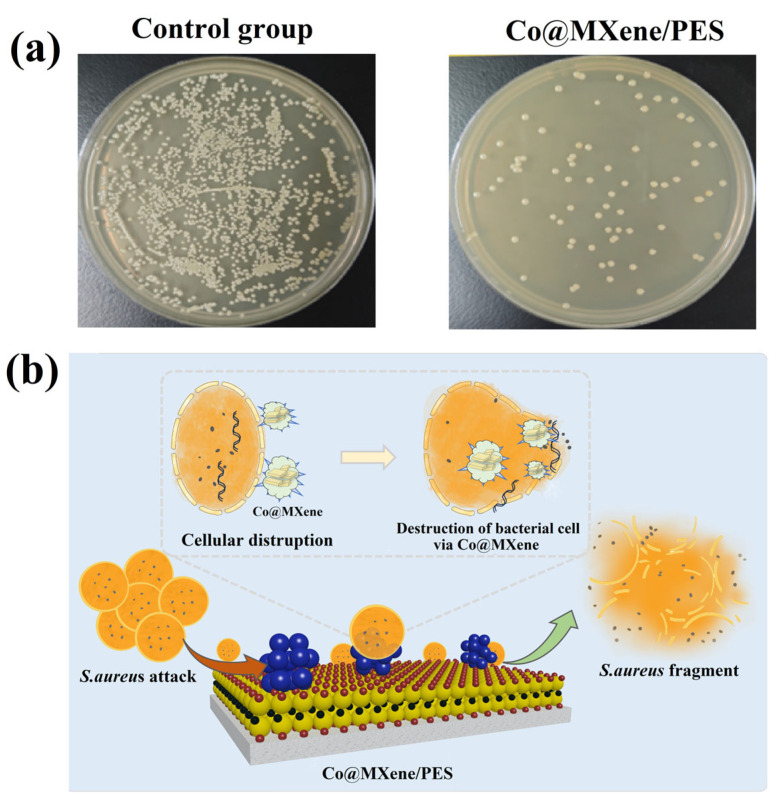
(**a**) Colonial growth of *S. aureus* on membrane and (**b**) schematic diagram of antibacterial mechanism.

In this study, *S. aureus* was used to investigate the antimicrobial properties of the Co@MXene/PES membrane. The growth and reproduction of the microorganisms is shown in Figure 7a. The mortality rate of bacteria in the Co@MXene/PES membrane was significantly increased to 95.10%. In addition, the schematic diagram of antibacterial mechanism is presented in Figure 7b. Currently, it has been shown that MXene nanosheets can kill bacteria by disrupting the bacterial membrane through direct interaction with bacteria, as well as induce oxidative stress to affect bacterial metabolism [41]. Meanwhile, cobalt nanoparticles (CoNPs) have been found to possess antibacterial properties. Since CoNPs can generate reactive oxygen species, which disrupt bacterial cell membranes and inhibit bacterial growth. The small size of the nanoparticles makes it easier for them to penetrate bacterial cells, thus increasing antibacterial activity [42]. Therefore, the synergistic effect between the simultaneous entry of MXene and CoNPs into the membrane surface can enhance the surface antimicrobial properties of the membrane and alleviate the phenomenon of membrane pore blockage caused by the growth of bacteria on the membrane surface during the application process.

### 2.6. Stability of the Catalytic Membrane

**Figure 8:**
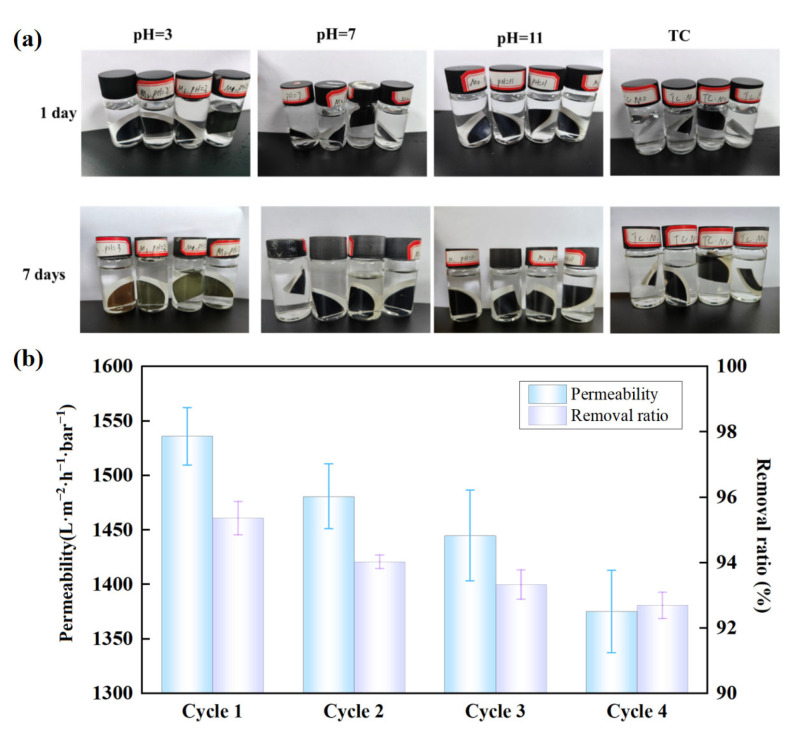
Stability and cyclic tests of membranes: (**a**) Digital photos of two-dimensional membranes after being immersed in solutions with different pH values for 1 day and 7 days (M0, M1, M2 and M3, from left to right); (**b**) The permeability and TC removal changes of the membrane under four cycles.

In practical applications, two-dimensional membranes can experience swelling, leading to increased interlayer spacing and possible detachment from the support layer. Therefore, resistance to swelling and reusability are key indicators of membrane stability. To assess the mechanical performance of the composite membranes, samples were cut into equal-sized pieces and immersed in strong alkaline, strong acidic, neutral, and TC solutions for seven days to evaluate their swelling properties. As shown in Figure 8a, the membranes exhibited excellent resistance to swelling under neutral and alkaline conditions, maintaining size stability and mechanical strength. In TC solution, the membranes demonstrated outstanding swelling resistance with stable structure and morphology. However, exposure to strong acidic solutions caused yellowing of the membrane surface, likely due to chemical reactions or oxidation of surface functional groups. Additionally, Figure 8b shows that while membrane permeability decreased after four cycles, the change was minimal, indicating good resistance to fouling and stable flux. Overall, the catalytic membranes displayed high stability and good reusability.

### 2.7. Degradation Mechanism

**Figure 9:**
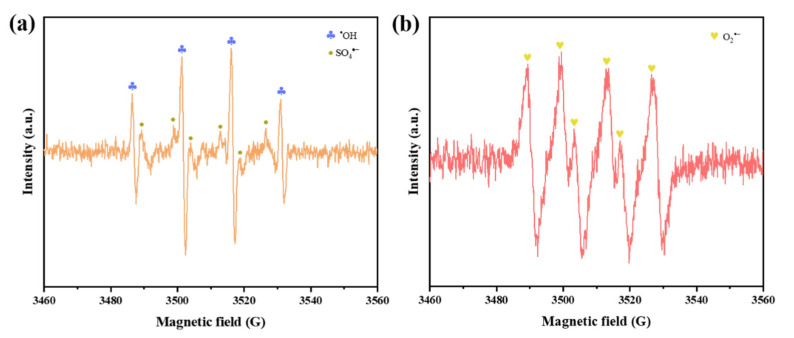
EPR spectra of (**a**) ^•^OH, SO_4_^•−^ and (**b**) O_2_^•−^.

**Figure 10:**
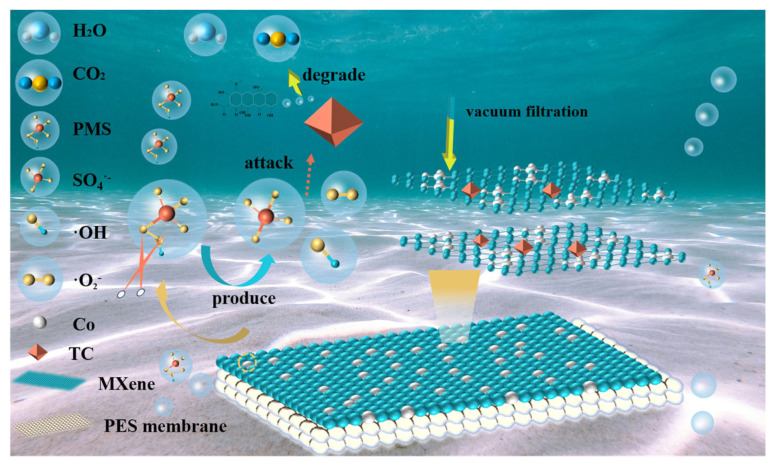
The degradation and separation mechanism of Co@MXene/PES membrane.

The Co@MXene/PES membrane, with its specific two-dimensional interlayer channels, physically filters out TC molecules while allowing water and smaller molecules to pass through. TC molecules are initially adsorbed onto the MXene membrane surface due to interactions with surface functional groups. To confirm the generation of ROS, electron paramagnetic resonance (EPR) experiments were conducted. The EPR detection signals using DMPO as the spin-trapping agent were shown in Figure 9. The signal with a peak intensity ratio of 1:2:2:1 confirmed the presence of ^•^OH in the reaction system. Although SO_4_^•−^ is difficult to detect due to its short lifespan and low sensitivity, a signal peak corresponding to SO_4_^•−^ could still be observed adjacent to the ^•^OH signal, indicating the presence of SO_4_^•−^ during the catalytic reaction process [43]. Additionally, a signal with a peak intensity ratio of 2:2:1:2:1:2, characteristic of O_2_^•−^, further confirmed the presence of O_2_^•−^ [44]. As shown in Figure 10, Co on the alkaline MXene surface acts as an active site for PMS activation. The activation of PMS generates SO_4_^•−^ and ^•^OH, which are crucial in the degradation process. SO_4_^•−^ reacts with TC to form intermediate products that are further oxidized by ·OH, gradually breaking down into simpler compounds. Through a series of oxidation reactions, most of TC is ultimately decomposed into non-toxic byproducts such as CO_2_ and H_2_O. This combination of physical sieving and chemical degradation effectively removes TC from water [45].

## 3. Materials and Methods

### 3.1. Preparation and Alkalization Modification of MXene

The reagents and materials used in this study are detailed in Supplementary Materials 1.1, while the methods and instruments for characterizing and analyzing nanomaterials and membranes are summarized in Supplementary Materials.

According to previous literature on MXene preparation [12,46], MXene (Ti_3_C_2_T_x_) was obtained by etching the MAX (Ti_3_AlC_2_) phase with a mixed solution of LiF and HCl, followed by alkali modification. At the beginning, the etched MXene was ultrasonically dispersed for 15 min. Then, 1 M NaOH solution was prepared, and appropriate amount of MXene was dissolved in the NaOH solution to obtain a dispersion with a concentration of 0.5 mg/mL. This dispersion was then treated in an ultrasonic cell crusher for 1–2 h. Subsequently, the mixed solution was centrifuged in a centrifuge machine at 10,000 rpm for about 10 min. This centrifugation process was repeated multiple times until the pH of the supernatant reached 7. Finally, the centrifuged precipitate was collected and dried in a vacuum drying oven at 60 °C for 12 h to obtain MXene-NaOH nanosheets.

### 3.2. Synthesis of Co-MXene Materials

The dried MXene-NaOH nanosheets were re-dispersed in deionized water to obtain a dispersion with a concentration of 0.5 mg/mL. After 2 h of ultrasonication, a certain amount of CoCl_2_ powder with Co atomic concentrations of 0, 0.005, 0.01, and 0.03 mg/mL was added proportionally. Subsequently, 5 mM of ascorbic acid (VC) was added, and the mixed solution was put into a water bath at 80 °C for 2 h. After cooling to room temperature, the prepared mixture was centrifuged, and the centrifuged precipitate was collected and subsequently put into a vacuum drying oven at 60 °C for 12 h. Finally, the Co-MXene powder was obtained.

### 3.3. Construction of Two-Dimensional Co@MXene/PES Membranes

**Figure 11:**
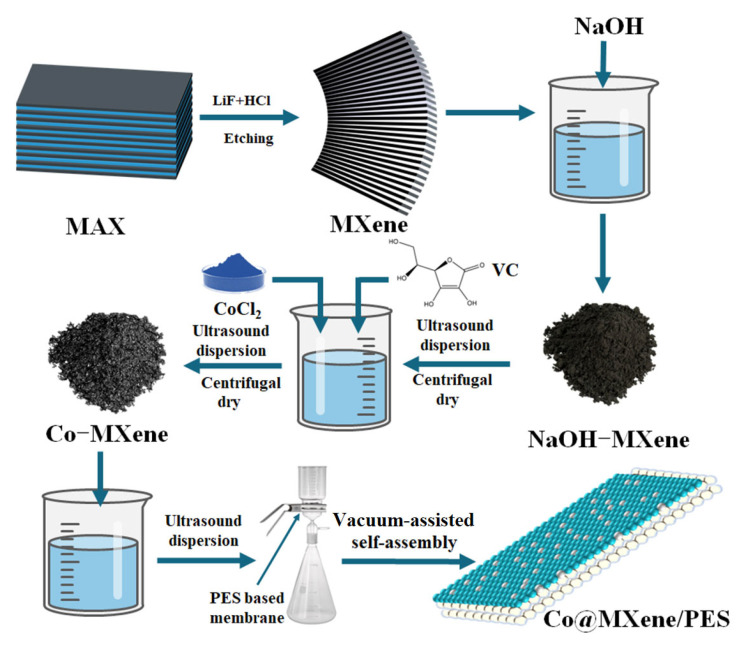
The fabrication steps of Co@MXene/PES membrane.

Firstly, 12 mg of Co-MXene powder was weighed and added to deionized water, and the mixture was sonicated for 30 min using an ultrasonic device to ensure uniform dispersion of the Co-MXene powder. After sonication, the Co-MXene dispersion was deposited onto the PES membrane substrate using a vacuum filtration device to obtain Co@MXene/PES membranes. The synthesis process of the Co@MXene/PES membranes is illustrated in Figure 11.

The composition of the membranes in different ratios is listed in Table 1.

### 3.4. Permeability and Separation Properties

In this experiment, the permeation and separation performance of the membranes were assessed using a dead-end filtration device under a pressure of 0.09 MPa. The pure water flux (*J*_w_), which indicates the membrane’s permeability, was measured. The flux of 100 mL of deionized water through the two-dimensional membranes was measured under the pressure of 0.09 MPa. Here, *V* represents the initial volume of the liquid (0.1 L), *A* denotes the effective membrane area (12.56 cm^2^ in this experiment), and *t* is the testing time. The flux can be calculated using Equation (1):

(1) $$J_{w}=\frac{V}{A\times t}$$

The separation performance of the membranes was tested using 100 mL of a TC standard solution (20 mg/L). *C_p_* and *C_f_* are the TC concentration in permeated and feed solutions, respectively. The TC rejection rate (*R*) was calculated according to Equation (2):

(2) $$R=\left(1-\frac{C_{P}}{C_{f}}\right)\times 100\%$$

### 3.5. Removal Capacity of the Antibiotics

A volumetric flask was used to prepare 500 mL of TC standard solution with a concentration of 20 mg/L. 100 mL of TC solution was transferred to a beaker, and 30 mg/L PMS solution was added. Then TC solution was then treated with the synergistic effect of two-dimensional Co-MXene membrane and PMS at 0.09 MPa. The absorbance *A*_0_ of the initial TC solution and the absorbance *A*_1_ of the treated TC solution were measured at 358 nm. The TC removal rate (*φ*) was calculated from Equation (3):

(3) $$\varphi =\left(1-\frac{A_{1}}{A_{0}}\right)\times 100\%$$

### 3.6. Antibacterial Activity Tests 

In this experiment, Gram-positive *Staphylococcus aureus* (*S. aureus*) was selected as a simulated microorganism to study the effect of the membrane surface on the normal growth and reproduction of bacteria before and after modification, concerning the literature of Dong et al. [47]. Firstly, the membrane was irradiated using ultraviolet light for 1 h in a sterile environment for thorough sterilization pretreatment. Then, an alcohol lamp was lit on the ultra-clean bench. The following operation was carried out in a sterile environment: 100 μL of bacterial suspension of *S. aureus* (the concentration of the bacteria was about (1.0~1.5) × 10^6^ cfu/mL) was placed into a centrifugal tube containing 20 mL of sterile physiological saline together with the membrane sheet and cultured in a constant-temperature incubator at 37 °C for 2 h. At the end of the reaction, 100 μL of the mixed solution was evenly coated on a sterile LB solid medium and incubated in a constant temperature incubator at 37 °C for 24 h. The number of colonies on the medium was recorded. At the same time, the number of colonies on the medium coated with the bacterial suspension not in contact with the diaphragm under the same culture conditions was recorded as the control group. The bacterial mortality rate was calculated using Equation (4):

(4) $$M=\frac{B-A}{B}\times 100\%$$

where *M* (%) is the bacterial mortality rate, *A* is the number of bacteria that survived contact with the antimicrobial membrane during the contact time, and *B* is the number of bacteria that survived without contact with the antimicrobial membrane but under the same conditions during the same incubation time (blank control).

## 4. Conclusions

In summary, this study successfully constructed Co@MXene/PES two-dimensional membranes using a vacuum filtration method and verified the impact of cobalt introduction on the MXene membrane through various characterization techniques. The experimental results demonstrated that the Co@MXene/PES membranes effectively catalyze the degradation of antibiotics through the activation of PMS. The findings indicate that cobalt atoms not only increase the interlayer spacing of MXene, thereby enhancing the flux of the membrane (up to 1584.60 L·m^−2^·h^−1^·bar^−1^), but also confer catalytic degradation capability to the membrane through the synergistic effect with PMS, achieving a degradation rate of up to 95.51% at a cobalt concentration of 0.01 mg/mL. Additionally, antimicrobial and stability tests of the membrane revealed excellent antibacterial properties (with a 95.10% death rate for *S. aureus*), outstanding anti-swelling performance in TC solutions, and minimal change in flux after repeated cycling. These results suggest that the Co@MXene/PES two-dimensional membrane not only enhances pollutant removal efficiency but also improves anti-fouling properties, making it suitable for reuse and recycling. This provides a novel approach for the efficient treatment of TC pollutants in water, ensuring long-term stable operation and safe water discharge.

## Figures and Tables

**Table 1:** The detailed composition of Co@MXene/PES membranes.

Membrane	The Concentration of Co Atoms	The Mass of Co-MXene
M0	0 mg/mL	12 mg
M1	0.005 mg/mL	12 mg
M2	0.01 mg/mL	12 mg
M3	0.03 mg/mL	12 mg

## Data Availability

The data that supports the findings of this study is available.

## Supplementary Materials

The following supporting information can be downloaded at: https://www.mdpi.com/article/10.3390/molecules29214995/s1, Figure S1: (a) FTIR spectra of MXene, NaOH-MXene, and Co-MXene; (b) XRD patterns of MXene, NaOH-MXene, and Co-MXene; Figure S2: (a) XPS total spectrum of M3; fine spectra of M3 for N1s (b), Ti2p (c) and Co (d). Table S1: The elemental composition of Co-MXene nanocomposite.
