# Supplementary material for: Construction of Co-Modified MXene/PES Catalytic Membrane for Effective Separation and Degradation of Tetracycline Antibiotics in Aqueous Solutions
Source: Molecules. 2024 Oct 22;29(21):4995. doi: 10.3390/molecules29214995 (PMC11547915; doi:10.3390/molecules29214995)
Supplement: Supplementary file 1 [file molecules-29-04995-s001.zip › molecules-3199378-supplementary.pdf]

Supplementary Materials

Construction of Co-Modified MXene/PES Catalytic Membrane for Effective Separation and Degradation of Tetracycline Antibiotics in Aqueous Solutions

Xiaojie Cheng [1,†,*], Xiaojun Qin [2,†], Runxue Zhao [1], Jiamin Chen [1], Xia Zheng [2], Ke Liu [2] and Meixuan Xin [2]

1. College of Life Science, Sichuan Normal University, Chengdu 610101, PR China; f872864996@163.com (R.Z.); eidolonamaz@outlook.com (J.C.)
2. College of Materials and Chemistry & Chemical Engineering, Chengdu University of Technology, Chengdu 610059, PR China; xjqin913@163.com (X,Q.); zhengxia@stu.cdut.edu.cn (X.Z.); 2023020668@stu.cdut.edu.cn (K.L.); mango_xinmeixuan@163.com (M.X.)

* Correspondence: Email: xjcheng1991@163.com (X.C.)

† These two authors contributed equally to this work

1. Supplementary Methods

1.1 Materials

MAX (Ti_3AlC_2) powder was provided from Jilin 11 Technology Co., Ltd. Lithium fluoride (LiF, 99%) and hydrochloric acid (HCl) were purchased from Chengdu Kelong Co., Ltd. NaOH was purchased from Chengdu Jinshan Chemical Reagent Co., Ltd. Peroxymonosulfonate (PMS) and $CoCl_2$ were purchased from Shanghai adamus reagent Co., Ltd. The L-ascorbic acid (VC) and Tetracycline hydrochloride (TC) were obtained from Shanghai shao far reagent Co., Ltd. and Shanghai Maclin biochemical technology Co., Ltd. respectively. The PES commercial membrane (d=50 mm, pore size=0.22 μm) was purchased from Jinteng Experimental Equipment Co., Ltd.

1.2 Synthesis of MXene nanosheets

First, 0.5 g of lithium fluoride were weighed and dissolved in 15 mL of 12 M hydrochloric acid. Then, 0.5 g of MAX phase were added to the solution, and the mixture was stirred with a magnetic stirrer at 30℃ for 20 h. After etching was completed, the mixture was centrifuged and washed with deionized water. The supernatant containing multi-layer MXene was treated under nitrogen protection for 8 h (preventing MXene from oxidizing into TiO_2), followed by another centrifugation. The collected supernatant was finally freeze-dried to obtain monolayer or few-layer MXene nanosheets.

1.3 Characterization

Fourier-transform infrared spectroscopy (FTIR) was employed to identify the types of functional groups within MXene and Co-MXene materials. X-ray diffraction (XRD) and X-ray photoelectron spectroscopy (XPS) was used to analyze the crystal structure and chemical composition of composite membrane. Scanning electron microscopy (SEM) was employed to observe the morphology and structure of the nanomaterials and composite membranes. Energy dispersive spectrometer (EDS) was used to analyze the elemental composition of different materials. Transmission electron microscopy (TEM) was utilized to determine the internal microstructure of the

materials. Atomic force microscopy (AFM) was used to characterize the surface roughness of the composite membranes.

2. Supplementary Figures

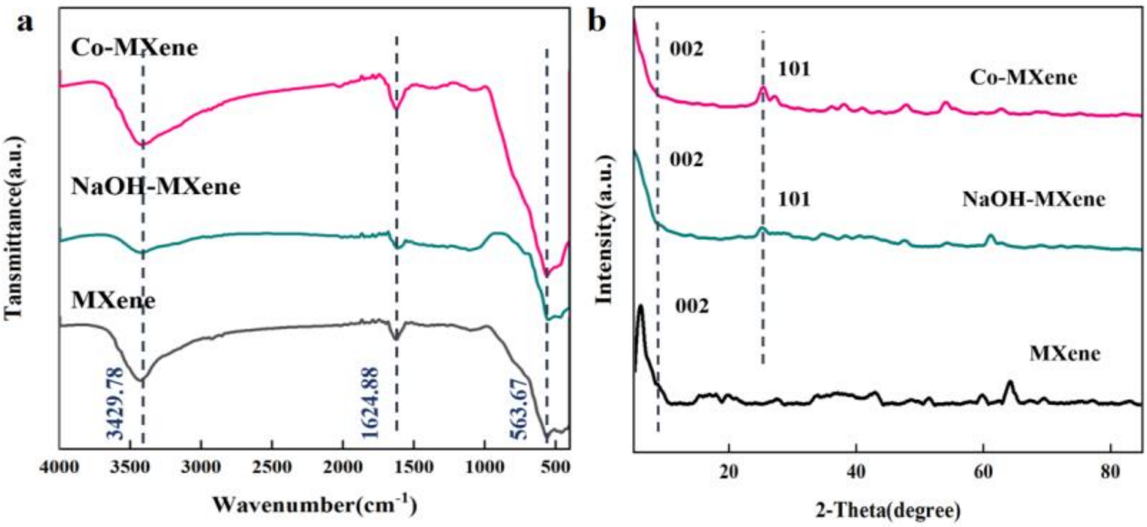

Figure S1. (a) FTIR spectra of MXene, NaOH-MXene, and Co-MXene; (b) XRD patterns of MXene, NaOH-MXene, and Co-MXene.

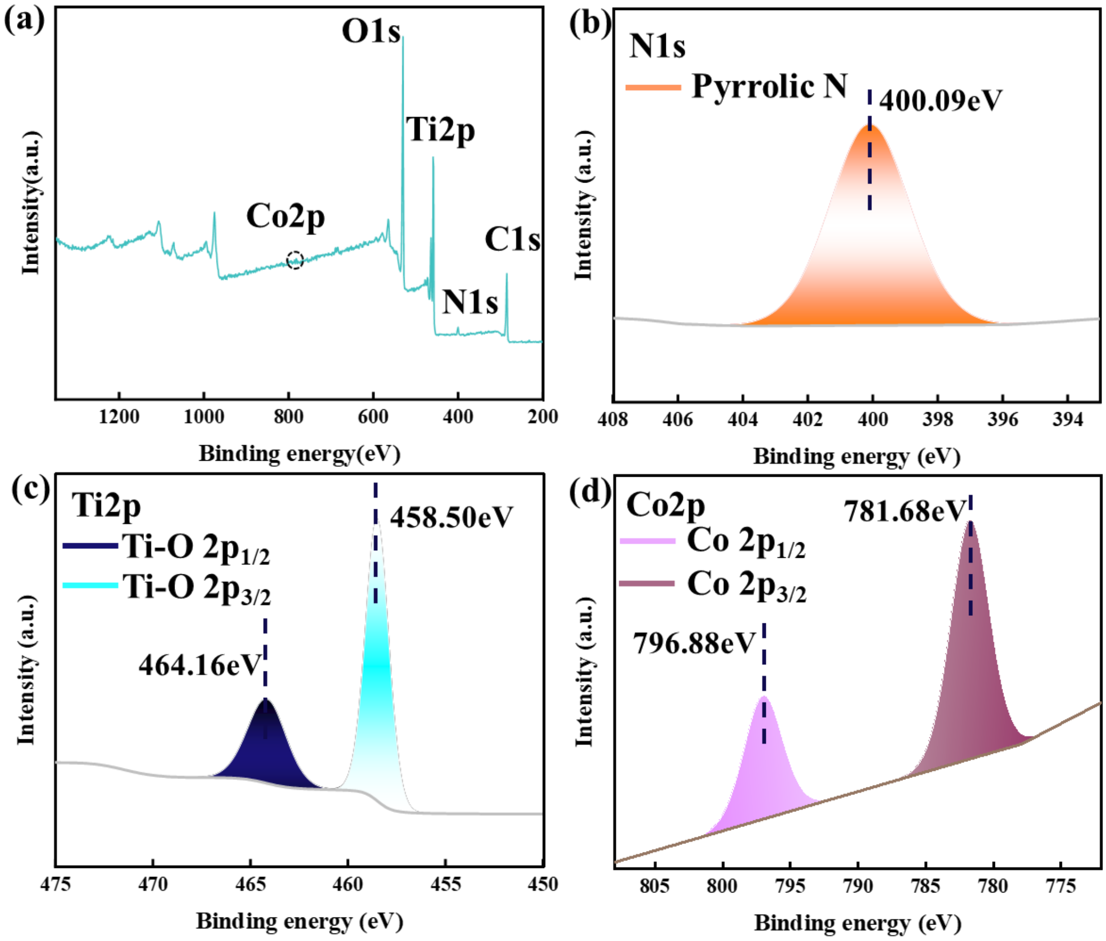

Figure S2. (a) XPS total spectrum of M3; fine spectra of M3 for N1s (b), Ti2p (c) and Co (d).

3. Supplementary Table

Table S1. The elemental composition of Co-MXene nanocomposite.

Element	Weight percentage	Atomic percentage	Intensity
C	33.2	52.6	55.3
N	0.8	1.07	1.1
O	25.5	30.3	41.3
Na	0.0	0.22	0.0
Ti	40.1	15.9	450.1
Co	0.3	0.81	0.1
